# Aqueous Dispersions of Esterified Lignin Particles for Hydrophobic Coatings

**DOI:** 10.3389/fchem.2019.00515

**Published:** 2019-07-18

**Authors:** Qi Hua, Li-Yang Liu, Muzaffer A Karaaslan, Scott Renneckar

**Affiliations:** Department of Wood Science, The University of British Columbia, Vancouver, BC, Canada

**Keywords:** hydrophobic materials, lignin, coating, organic acid, sustainable, microparticles, wax, nature-inspired

## Abstract

An aqueous biopolymer dispersion coating system was synthesized utilizing softwood kraft lignin and a long chain organic acid. The chemical treatment of lignin was a two-step procedure, which first consisted of hydroxyethylation of the phenolic groups on lignin utilizing ethylene carbonate and an alkaline catalyst. This first step resulted in the lignin containing more than 80% aliphatic hydroxyl functionality (^1^H NMR). Following this step, oleic acid was reacted with hydoxyethyl lignin in order to form ester derivatives. With nearly a total reduction in absorbance in the hydroxyl stretching region, FT-IR analysis showed the majority of the hydroxyl groups was esterified forming an ethyl oleate derivative. Semi-quantitative ^13^C NMR analysis of the lignin revealed 88% substitution of the lignin hydroxyl groups. This derivative was soluble in organic solvent such as toluene and tetrahydrofuran. Solutions of lignin derivatives were slowly precipitated through dialysis, resulting in a stable dispersion of lignin microparticles in distilled water. The 1–2 μm average diameter size of the precipitated particles was found with dynamic light scattering of the suspensions. Spray and spin coating were used to apply the lignin derivative dispersion to different surfaces. For both coating methods, the lignin-based particles enhanced the hydrophobicity of all the substrates tested, resulting in increased water contact angles for glass, kraft pulp sheets and solid wood. Benign reagents involved in the coating synthesis utilized natural compounds that are known to repel water in nature. Combined with the avoidance of volatile organic solvents during application, this process provided a low environmental footprint solution for synthesis of hydrophobic coatings.

## Introduction

Wood-based products are an important class of materials in modern society with the ability to contribute to a more sustainable world (Mohanty et al., 2002). Wood based renewable materials can span from the nanoscale (Jiang and Hsieh, 2014; Foster et al., 2018; Jiang et al., 2018), to modify properties, to macroscale structures that perform even better than many metal alloys. Further, new building codes provide greater opportunities for wood-based materials in tall wooden structures with designs toward wooden skyscrapers (Mohammad et al., 2018). While wood structures provide more design opportunities, perceptions that surround longevity of wooden structures, such as fire, pests, and decay, limit further consideration of wood in many applications (Marney and Russell, 2007). Timbers can biodegrade in high-moisture environments, under ambient conditions if the cell wall is saturated with water; moisture above this fiber saturation point of the cell wall enables mold and decay fungi to attack the structure of the cell wall, depolymerizing the polysaccharides into simple sugars (Eriksson et al., 2012). For wood materials, loss of mass by just a few percent caused by decay fungi, significantly impacts the toughness of wood (Gordobil et al., 2017; Mohanty et al., 2018). Hence, durability impacts the overall performance and moisture exposure can occur in service or during handling and storage at the construction site.

In nature, trees with protection from external factors can survive hundreds of years. In trees, phloem tissue serves as protection from fungi spores and bacteria; a significant fraction of this phloem composition is suberin, a polymeric compound that is a combination of aromatic and aliphatic structure (Franke and Schreiber, 2007). On external surfaces such as leaves, a variety of long chain aliphatic organic compounds in epicuticular waxes imparts hydrophobicity to its surfaces. Highly structured waxes with microscale structures and nanoscale textures can create superhydrophobicity on the leaves of plants (Hu and Deng, 2010). Hence, the use of aliphatic carbon chains is a simple approach to make highly hydrophobic materials. Recently, this method was recreated utilizing cellulose derivatives to produce hydrophobic coating materials (Geissler et al., 2013). Geissler and co-workers developed superhydrophobic coating materials from stearic esterified micro-cellulose particles. Results showed that the micro-/nano scale particle geometries could significantly improve the hydrophobicity of the surface and develop self-cleaning properties, mimicking the lotus leaf.

In addition to nature inspired approaches to limit water absorption and improve water resistance of wood-based materials, chemical methods can directly block hydroxyl groups within the cell wall. Chemical treatment by acetylation or other hydrophobizing agents improves the water resistance of wood materials, but a large amount of reagents is necessary during this process (Lv et al., 2018), and the samples typically require some type of pressurized reactor. These treatments are appropriate for wood-based materials in direct exterior exposure. However, in most cases, a short-term storage needs or temporary protection during construction requires a low-cost treatment solution for lumber or composite panels.

For coatings, renewable materials utilizing biopolymers, such as lignin, were previously explored. Hult et al. determined that esterified lignin showed improved water-proofing for paper board (Hult et al., 2013). Lignin derivatives have also been used to coat wood surfaces to improve hydrophobicity as exhibited with reduced wettability measurements (Gordobil et al., 2017). For these samples, the esterification process was shown to increase the hydrophobic nature of lignin. Halogenated or anhydride compounds were adopted in this process with either partial and full conversion of lignin into its esterified form (Salanti et al., 2016). These methods were performed under standardized conditions requiring solvent, which increases the environmental factor or “E-factor”; the E-factor is a metric that quantifies the amount of product to the amount of waste generated (Sheldon, 2007). The methods of lignin esterification leading to hydrophobization, typically reported in the literature utilized halogenated or anhydride compounds that potentially cause negative environmental issues. For these cases, the green chemistry principle of the use of renewable feedstocks may be offset by creation of waste and toxic byproducts (Gillet et al., 2017). Further, hydrophobic coating applications usually require toxic or highly volatile solvents such as chloroform or acetone for the application of these hydrophobized bio-based coatings (Urushihara and Nishino, 2005). These coatings release volatile organic compounds from the solvent during application, which potentially will cause health hazards with repeated exposure (Clark et al., 2015). In order to avoid these problems, direct esterification was developed by Liu et al using benign and low-cost organic acid as solvent and reagent to efficiently modify the aliphatic OH groups in lignin with carbon length 3~18 (Liu et al., 2019).

Working from this approach, softwood kraft lignin (extracted from LignoForce system) (Kouisni et al., 2012) was modified utilizing a two-step direct esterification to obtain an ethyl oleate lignin. Specifically, the lignin was modified by ethylene carbonate to obtain lignin only containing aliphatic hydroxyl groups (Liu et al., 2018). Then oleic acid was utilized as both solvent and reagent to realize the direct esterification of hydroxyethyl lignin derivative. This technique created both an aromatic and aliphatic carbon component that is a simplified model of suberin material, found in bark, for the protection of wood. The obtained esterified lignin was then prepared as aqueous micro-lignin particles via dialysis to use as a hydrophobic thin coating for wood-based materials.

## Materials and Methods

### Material and Chemical Reagents

Softwood kraft lignin (SKL)AQQ7 Amallin A™ was donated by West Fraser Corp. (Hinton, Canada). The original SKL had 40% moisture content and was slightly acidic (pH = 2, 15% solid lignin in water). These lignins were then thoroughly washed with distilled water until to a neutral pH was attained and dried by lyophilization before the modification and characterization.

Ethylene carbonate (EC, Alfar Aser), sulfuric acid (Thermal Fischer), oleic acid (Sigma Aldrich), tetrahydrofuran (HPLC grade, Sigma Aldrich) were used, as received. Deuterium reagents including chloroform-D were purchased from Cambridge Isotope Laboratories, Int. Dimethyl sulfoxide-d_6_ and toluene -d_8_ were obtained from Sigma Aldrich. Anhydrous pyridine was prepared by adding molecular sieves in the Pyridine (Sigma Aldrich, 99.8%).

### Preparation of Hydroxyethyl Lignin (HELignin)

Twenty grams of dried lignin powder was load into a 500 ml round bottom flask. Based on a molar ratio to the aromatic hydroxyl group (ArOH, 3.6 mmol/g) in lignin, 64 g ethylene carbonate (10 ArOH) and 2.4 g Na_2_CO_3_ (0.1 ArOH) were added. The flask was covered with a rubber septum held in place by wire. After purging nitrogen gas to remove air, the reaction proceeded in an oil bath under 120°C for 2.5 h. At the end of the reaction, the solution was poured slowly into 400 ml pH = 2 aqueous sulfuric acid with continuous stirring to precipitate modified lignin. The mixture was then filtered by 0.45 μm PVTF membrane, followed by washing with another 400 ml distilled water until neutral pH and dried by freeze dryer (Liu et al., 2018).

### Esterification of HELignin

The direct esterification (catalyst-free esterification) was adopted to obtain esterified lignin following our previous procedure (Liu et al., 2018, 2019). Specifically, 3 g hydroxyethyl lignin was mixed with 30 ml oleic acid in a round bottom flask, which was heated under 180°C (oil bath) for 24 h. After cooling down, the samples were mixed with filter aids (Celite 545, Sigma Aldrich) and washed by 300 ml aqueous ethanol (75% v/v). The mixture was then filtered by 0.20 μm PVTF filter membranes. The lignin-based ester was then washed with 75% aqueous ethanol twice and distilled water twice. THF was used to dissolve the ester product, separating it from solid filter agent by filtration across Whatman No.1 filter paper. THF was added to the remaining solid part and filtered. This step was repeated until the solid filter aid became white, to ensure all the ester product was dissolved by THF. Air dried lignin derivatives from THF were washed with ethanol and distilled water sequentially. The remaining esterified sample was freeze dried (Liu et al., 2019). The above esterification procedures were repeated with acid catalyzed reaction with 1% sulfuric acid (wt) included during the reaction. All reaction conditions and isolation procedures remained the same.

### Characterization of Lignin

#### Nuclear Magnetic Resonance Analysis

^31^P NMR analysis was used for the characterization of hydroxyl groups in lignin. Specifically, the solvent used to dissolve lignin samples was prepared prior, by mixing chloroform-D and pyridine in a ratio of 1: 1.6. Twenty milligram lignin samples were added into 400 μL of above solvent and then vortexed thoroughly until all solids were fully dissolved. Then the other compounds were added including 100 μL of internal standard (9.6 mg N-hydroxy-5-norbornene-2,3-dicarboximide in 1 ml above solvent), 40 μL of relaxation reagent (5.6 mg chromium (III) acetylacetonate in 1 ml above solvent), and 50 μL phosphorous reagents (2-chloro-4,4,5,5-tetramethyl-1,3,2-dioxaphospholane) (Argyropoulos, 1995; Pu et al., 2011).

^13^C NMR semi-quantitively analyzed the major aromatic groups, functional groups, and chemical linkages in lignin. Due to the different solubility of resulting lignin, deuterium dimethyl sulfoxide (DMSO-d6) was used to dissolve lignin and HELignin, and toluene-d8 was used for oleate HELignin. Specifically, 150–160 mg lignin samples were dissolved into 450 μL corresponding solvent, following with the addition of 60 μL relaxation reagent (50 mg/ml chromium (III) acetylacetonate in DMSO-d_6_ or toluene-d_8_) and 15 mg internal standard (1,3,5 trioxane) were added (Balakshin et al., 2016).

All above lignin solutions were transferred into 5 mm NMR tubes and tested by Bruker Avance 300 MHz equipped with a BBO probe at 25°C. Applied parameters for ^13^C NMR: scan number 20 000, relaxation delay 2 s, acquisition time 1.4 s, and pulse length 8.5 μs; ^31^P NMR: scan number 800, relaxation delay 5 s, pulse length 6 μs, and acquisition time 1.4 s.

#### FT-IR Analysis

Sample pellets for transmission FTIR were prepared by mixing 2–3 mg lignin with 200 mg of dried potassium bromide. After thoroughly mixing and compression, the lignin pellets were analyzed using the Perkin Elmer Infrared Spectroscopy (MA, United States). Spectra were collected with a resolution of 4 cm^−1^ and averaged over 32 scans.

#### Gel Permission Chromatography (GPC)

Five milligram acetylated lignin, acetylated HELignin, or oleate HELignin were dissolved into 1 ml tetrahydrofuran (THF), respectively. The acetylation procedure followed our previous study (Liu et al., 2018). The lignin solutions were stabilized for 48 h before the analysis. Lignin solution was analyzed utilizing Agilent 1100 GPC equipped with three columns including Styragel HR1, HR3, HR4 (Waters Corp.) and analyzed by differential refractive index detector (dRI, Wyatt Corp.) at temperature 35°C. THF was used as eluent at a flow rate 0.7 ml/min. The data was collected utilizing Astra 6.0 and analyzed by conventional calibration methods. The standard polystyrene with different molecular weight was used as standard curve (Lange et al., 2016).

#### Differential Scanning Calorimetry (DSC)

Seven milligrams of lignin was accurately weighed and sealed in aluminum pans. TA Q1000 (Thermal Analysis Corp.) machine was adopted to analyze the sample under nitrogen gas environment. An annealing procedure was run in advance: room temperature to 105°C with rate of 10°C/min. Samples then were cooled down to −50°C with rate of −5°C/min and then heated to 150°C with rate of 10°C/min, again. The glass transition temperature was analyzed based on the third cycle (Cui et al., 2013).

### The Preparation of Lignin Micro-/Nano- Particle Solution

Four hundred milligram lignin and oleate HElignin were dissolved in 20 ml THF, respectively. The solutions were then transferred into dialysis tubing (cellulose membrane, Sigma-Aldrich) and placed into 1,500 ml distilled water for 7 days under slow stirring (Figure S1); the distilled water was changed every 24 h. The final concentration for the coating solution was 20 mg/ml (Zhao et al., 2016).

### Dynamic Light Scattering (DLS)

After analyzing the distilled water as background, 1.5 ml aqueous lignin-particle solution was diluted by 600 ml of distilled water and stirred at 1,500 rpm for dynamic light scattering analysis. The data was collected and analyzed by Mastersizer 2000. Each sample was run in duplicate.

### Spray and Spin Coating

Three types of surfaces were selected for coating analysis: glass, kraft pulp sheets, and wood (yellow poplar). The glass slides were cleaned by sonication for 30 min to remove all contamination and the surfaces were dried with nitrogen gas. Lignin particle solution was added into an air brush reservoir for spray coating at a distance 25 cm. The Laurell WS 650 Spin Coater was used for spin coating at room temperature. The coated materials were dried under ambient condition in fume hood overnight. To build the coating, this process was repeated 10 times for both spin coating and spray coating.

### Contact Angle Analysis

Five microliter distilled water droplets were dispensed on different types of treated surfaces from a height of 2 mm. The contact angle was recorded using a highspeed camera. The initial contact angle was measured and analyzed by Drop Shape Analyzer (DSA) 1.9 software.

### Micromorphology Analysis

Scanning electron microscopy (SEM) images were obtained using Hitachi S-2600 Variable Pressure SEM (Tokyo, Japan) at10 kV. A drop of aqueous lignin particle solution was applied to the surface of aluminum film and dried in the fume hood overnight. Untreated and spin coated paper board were also prepared. Before the measurement, a layer from palatium/gold of 10 nm was coated on the surfaces of the samples.

Atom force microscopy images were taken with the Multimode AFM Nanoscope-III (Veeco Instruments, Santa Barbara, USA) using the ScanAsyst TM tapping mode. Four hundred microliter lignin micro particle solution was dropped on freshly cleaved mica, spin coated, and left in a fume hood overnight. AFM images were captured using RTESPA, Veeco Inst. With a silicon cantilever, scan rate of 0.383 Hz and a scan size of 20 × 20 μm (Cho et al., 2017).

## Results and Discussion

### A Two-Step Route Toward Esterified Lignin

**Scheme 1:**
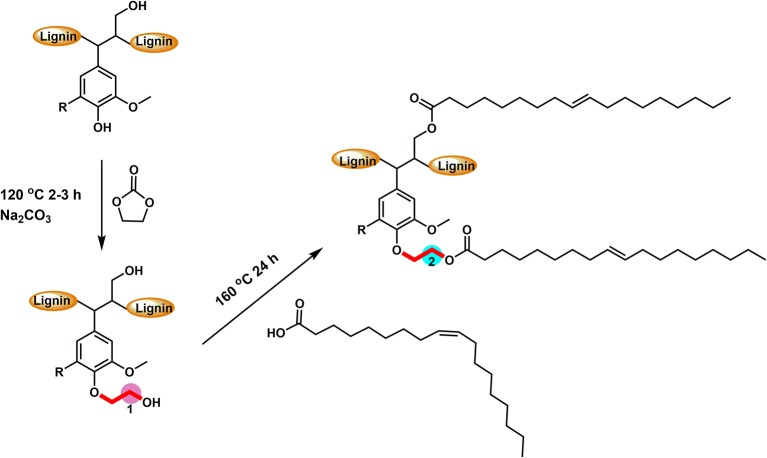
The reactive scheme of two-step esterification for the preparation of oleate hydroxyethyl lignin.

Softwood kraft lignin (SKL) contains different types of hydroxyl groups, which were reported to have different reactivity for esterification (Thielemans and Wool, 2005). Our previous research showed that the direct esterification, using organic acids as solvent and reagent, could successfully modify the aliphatic OH groups (Liu et al., 2019). Therefore, the hydroxyethylation with ethylene carbonate as both solvent and reagent was used to convert the aromatic OH groups into aliphatic OH groups (Scheme 1), to improve the relative reactivity of lignin toward organic acids (Liu et al., 2018). Figure S2 showed that the starting SKL has aliphatic OH (AlOH, peak 2, 150–145 ppm), aromatic OH (ArOH, peak 3, 144–136 ppm), and carboxylic acid groups (COOH, peak 4, 136–133 ppm) prior to the modification. After the hydroxyethylation, the ArOH and COOH groups were reduced from the spectrum, which signified that these compounds were modified. The exact values of each type of OH groups before and after the hydroxyethylation were reported in Table 1. For the hydroxyethylated lignin, the amount of AlOH increased from 2.07 mmol/g to 4.27 mmol/g, while the amount of total OH group decreased from 6.45 mmol/g to 5.03 mmol/g. The significant reduction of the ArOH (0.69 mmol/g) indicated that HELignin had a more uniform chemical structure with more than 80% of the ArOH converted into AlOH groups.

**Table 1:** Characterization of original lignin and hydroxyethyl lignin (HELignin); For ^13^C NMR, the aromatic carbon region (160–100 ppm) was set as 100 Ar.

	**AlOH** **(mmol/g)**	**ArOH** **(mmol/g)**	**COOH** **(mmol/g)**	**Total OH** **(mmol/g)**	**G** **/100Ar**	**MeO** **/100Ar**	**DC** **/100Ar**	**M_**w**_** **kDa**	**M_**n**_** **kDa**	**PDI**	**T_**g**_** **(^**°**^C)**
Lignin	2.07	3.87	0.56	6.49	80	83	50	6.184	1.366	4.53	165.1
HELignin	4.27	0.69	0.07	5.03	78	78	61	8.468	1.258	6.73	120.1

* *aliphatic OH groups (AlOH), aromatic OH groups (ArOH), carboxylic acid groups (COOH), guaiacyl units (G), methoxy content (MeO), degree of condensation (DC), degree of substitution (DS), average molecular weight (M_w_), Mn number average molecular weight (M_n_), polydispersity index (PDI), glass transition temperature (T_g_)*.

**Figure 1:**
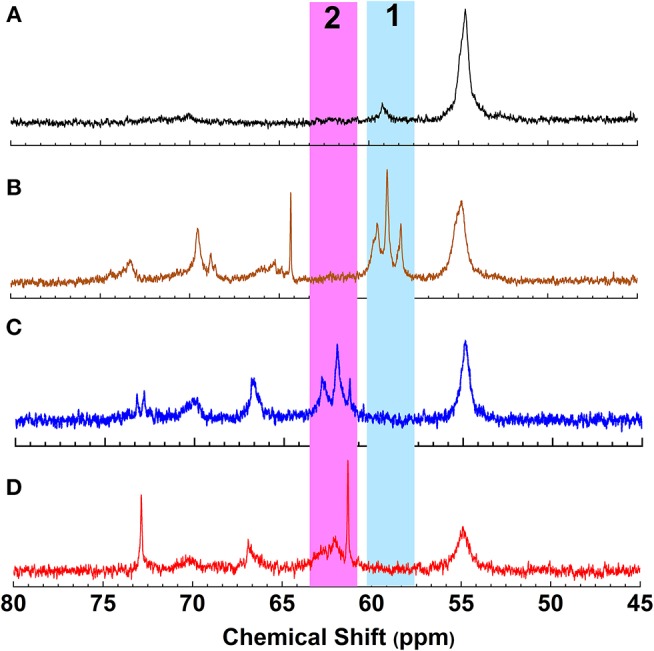
^13^C NMR spectrum of **(A)** Lignin, **(B)** Hydroxyethyl lignin (HELignin), **(C)** oleate HELignin, and **(D)** oleate HELignin with 1% H_2_SO_4_.

To achieve a lignin sample with hydrophobic characteristics, oleic acid was used to modify the aliphatic OH groups in lignin. Oleic acid was reported as a major component in plant oils potentially serving as abundant resource for making renewable materials (Zuo et al., 2011; Zhang et al., 2017). In order to obtain esterified lignin, high temperature reactions were required to promote the esterification process and providing enough hydrogen ions. To make a comparison, the Fischer esterification process which used 1% H_2_SO_4_ (98% conc.) as catalysts was also performed at the same temperature and reaction time. Based on ^13^C NMR spectrum, the degree of substitute (DS) for these two types of esterification routes was calculated based on the change of AlOH groups (*peak cluster 1*: 61.5–58 ppm, Scheme 1, Figures 1A,B), which was formed during the hydroxyethylation (Balakshin et al., 2016). After the esterification, the *peak cluster 1* disappeared, and the shielding of additional ester group formed a new peak region (*peak cluster 2*: 65–61.5 ppm, **scheme 1**). The degree of substitution (DS) for the esterification reaction was analyzed utilizing these peak structures: DS = peak 2/(peak 1+peak 2). The DS for the esterification with and without 1% H_2_SO_4_ were 89 and 88%, respectively (Table 2). Noteworthy, the peak 2 in Figure 1D (with H_2_SO_4_) had different peak shapes with *peak cluster 1* in Figure 1B and *peak cluster 2* in Figure 1C. This indicated that the addition of H_2_SO_4_ may have caused side reactions including the degradation of linkages or further condensation of lignin.

**Table 2:** Degree of substitution (DS), molecular weight, and glass transition temperature (T_g_) of oleate lignin without and with 1% acid.

	**DS** **%**	**M_**w**_/** **kDa**	**M_**n**_/** **kDa**	**PDI**	**T_**g**_** **(^**°**^C)**
Oleate HELignin	88	7.53	2.927	2.58	8.1
Oleate HELignin (1% acid)	89	7.096	0.815	8.71	2.7

**Figure 2:**
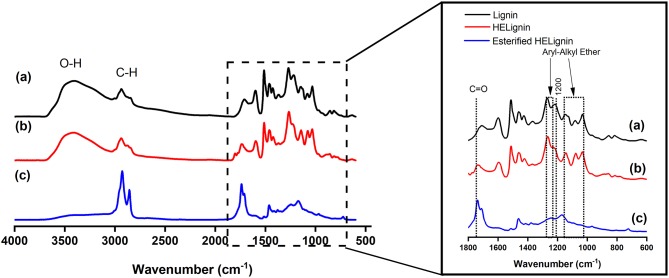
FT-IR spectrum of **(a)** Lignin, **(b)** Hydroxyethyl lignin (HELignin), and **(c)** oleate HELignin.

After the hydroxyethyl reaction, FT-IR spectrum showed that modified lignin had slight changes on Aryl-Alkyl ether bond and carbonyl groups (C = O, 1,800–1,600 cm^−1^) (Figure 2). Overall, there were few changes on the HELignin, as the hydroxyethyl reaction only selectively modified the aromatic OH groups without causing obvious side reactions. Confirming this change, ^1^H NMR spectrum showed the formation of hydroxyethyl group (3.8–4.5 ppm, Figure S4) in resulting HELignin

FT-IR analysis is a powerful method in revealing a change in hydroxyl stretching associated with these hydrophilic functional groups. In the spectrum there was evidence of the efficiency of our direct esterification (catalyst-free esterification) toward the hydroxyl groups in HELignin, as illustrated by a clear reduction of hydroxyl group (O-H stretching, 3,650–3,200 cm^−1^) absorbance. Further, there was evidence of the alkyl chains (C-H, 3,200–2,800 cm^−1^) and carbonyl linkages (COOR, 1,800–1,700 cm^−1^) (Figure 2). Proton NMR was also used to confirm modification. The attached alkyl chains in HELignin could be found in the upfield (1–2 ppm) of ^1^H NMR spectrum of oleate HELignin (Figure S4c). Overall, the hydrophilic hydroxyl groups in lignin were converted to hydrophobic alkyl chains. The obtained lignin-based ester derivative after the two step process, had a waxy chemical structure, which was used as a precursor for hydrophobic coating materials.

### Molecular Weight Analysis of Esterified HELignin

**Figure 3:**
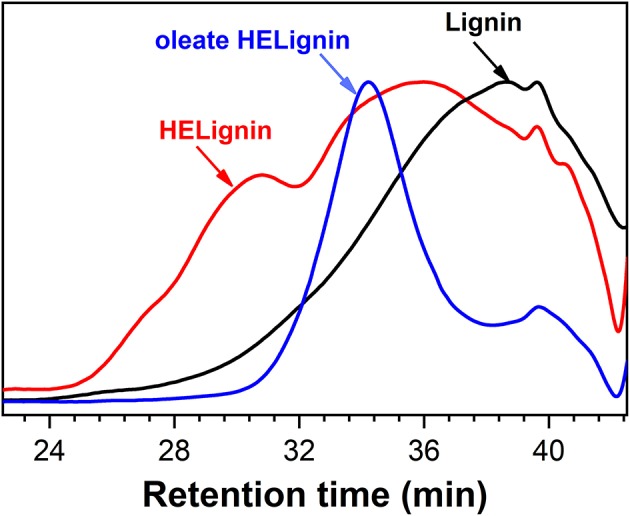
Gel permission chromatography traces of Lignin, Hydroxyethyl lignin (HELignin), and oleate HELignin.

The molecular weight was analyzed with gel permission chromatography (GPC) as shown in Figure 3. In agreement with our previous research on other lignin sourced materials, the HELignin had a slightly larger molecule weight (M_w_) and wider polydispersity index (PDI) (Figure 3). In addition to the hydroxyethyl groups on lignin, an increase in the degree of condensation was found arising from heating lignin in an alkaline environment. Based on the ^13^C NMR analysis, the degree of condensation increased from 50 (per 100 Ar) to 61 (per 100 Ar) during the hydroxyethyl reaction (Table 1). The degree of condensation was calculated based on the equation DC = 200+G-ArH (Figure S3) (Balakshin et al., 2016).

After the direct esterification, GPC analysis revealed that oleate HELignin with added acid catalyst had a much wider distribution of molecular weight and the traces had multiple peaks due to side reactions such as the condensation or degradation of ether bonds (Figure S5). As such, the lignin was not further considered for formulation of the hydrophobic coating. The esterified lignin via direct esterification without acid catalyst had a GPC trace revealing a more uniform molecular weight distribution than the other types of lignin (Table 2). However, the reduction of molecular weight for this lignin may be an artifact of the washing procedure to prepare materials for analysis.

### Thermal Properties of Esterified HELignin

The thermal properties of lignin before and after modification were also analyzed utilizing differential scanning calorimetry (DSC). The glass transition temperatures (T_g_) were detected based on the heat flow baseline shift after the third cycle in a heat-cool-heat program. By derivatizing the sample, the T_g_ was reduced from 165.1°C (SKL) to 120.1°C (HELignin). This reduction was larger than previously reported hydroxyethylated lignin sample that had first undergone acetone fractionation (Liu et al., 2018). The HELignin contained a higher amount of hydroxyethyl groups, which potentially would provide additional bulking to the lignin structure, reducing the T_g_ further. The ethyl-oleate derivative had a significant change of T_g_ before and after the reaction; reducing the T_g_ from 120.1°C down to 8.1°C or 2.7°C (1% H_2_SO_4_ as catalyst) (Table 2). This result occurred because the esterification reaction had significantly reduced the amount of aliphatic OH groups (more than 4 out of 5 hydroxyl groups were modified with the long carbon chains), which directly weakened the intra and intermolecular bonding network and caused an increase in free volume. Hence, the alkyl chains created bulky side-groups that enhanced the inner plasticization of the lignin derivative. The change on the T_g_ of esterified lignin was similar with previous report that using halogenated compounds to obtain a series of lignin with different carbon chain lengths. The T_g_ of esterified lignin was reduced with increasing carbon chain length and the degree of substitution (Koivu et al., 2016).

### Aqueous Lignin Particle Solution

**Figure 4:**
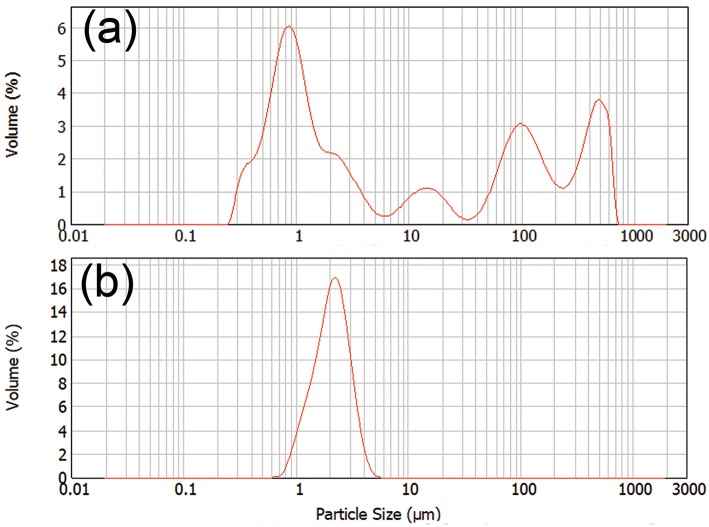
The particle size distribution of **(a)** Lignin, **(b)** oleate HELignin.

Dynamic light scattering was used to characterize the particle size of the dispersion after controlled precipitation. Figure 4 showed the distribution of particle sizes of SKL and the oleate HELignin derivative. The particle size for SKL had a large distribution in a range from 200 nm up to the maximum detectable range. This large range of particle sizes may be related to the swelling of the lignin via the available hydroxyl groups. The lignin formed large particles and were precipitated after extended storage. Conversely, the esterified lignin particle solution formed a steady dispersion with a particle size centered at 2 μm. The dispersion was stable for more than 1 week without precipitation.

### Contact Angle Analysis

The target application of this lignin-based material was to serve as a sprayable water-based renewable material coating for forest-based products, which is critical in construction and packaging (Gordobil et al., 2017). To illustrate the change in surface behavior, the wettability of the modified surface was investigated using a static contact angle test. Bleached kraft pulp sheet stock, wood, and glass were selected as the test substrates to investigate the hydrophobicity of the coatings consisting of the lignin-oleate microparticles. The oleate esterified HELignin suspension was sprayed onto the various surfaces utilizing an airbrush. Multiple coating cycles were used to form a uniform layer. However, uniformity was difficult to achieve on the non-porous surface. The spin coating was adopted to form a uniform layer on the surface of target materials.

**Figure 5:**
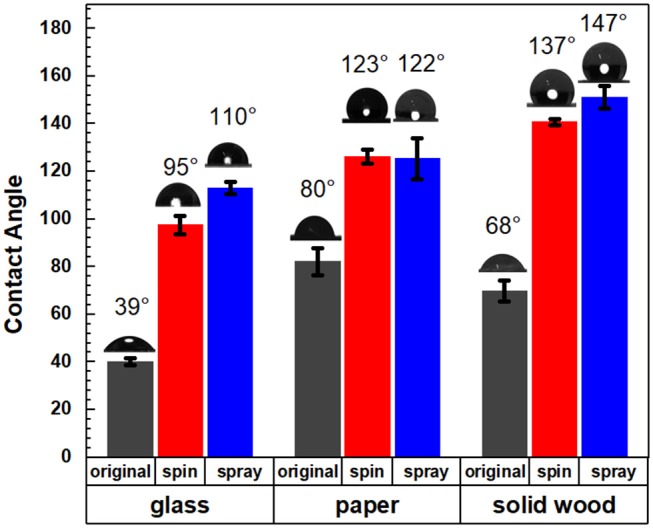
Contact angles of original and coated glass, paper, and solid wood with spin and spray coating methods.

Figure 5 showed that the coating suspensions would increase the hydrophobicity for all three types of material surfaces for both spray and spin coating. The lignin ester deposited on the glass slides had a contact angle 110° (spray) and 95° (spin), while uncoated samples had an angle of 39°. The aqueous lignin particle solution was applied to two common wood-based products which included a solid wood block of yellow poplar and a kraft pulp sheet. For the solid wood, the contact angle increased from 68 to 147° (spray) and 137° (spin) after coating with the lignin derivative. This value is more than 20 and 10° greater than previous reported values for organosolv hydrophobized lignin esters on poplar (Gordobil et al., 2017). For the pulp sheet, the contact angle increased significantly from 80° to 122°(spray) and 123°(spin). The supporting video showed that the water droplets disappeared in 10 s for uncoated pulp, while the water droplets on the coated materials were stable after more than 2 min (Videos S1, S2). Further, the initial contact angles of the lignin oleate ester coated handsheet were similar to reported values for coated filter paper or coated commercial paper board with suberin-like lignin ester derivatives (Antonsson et al., 2008) or tall oil lignin ester derivatives (Hult et al., 2013), respectively. In these cases, the process required dissolution of the lignin derivative into acetone and application to the substrate with an organic solvent. In contrast, spraying or spinning from an aqueous suspension avoids potential VOCs during the application process.

Research into lignin-based particles (Abbati de Assis et al., 2018) is of growing interest from emulsions (Ago et al., 2016) to surfaces (Cusola et al., 2018). This research offers a novel route to tune the chemistry of the lignin particles in a very simple method of direct esterification.

### Micromorphology Analysis of Aqueous Lignin Coating

**Figure 6:**
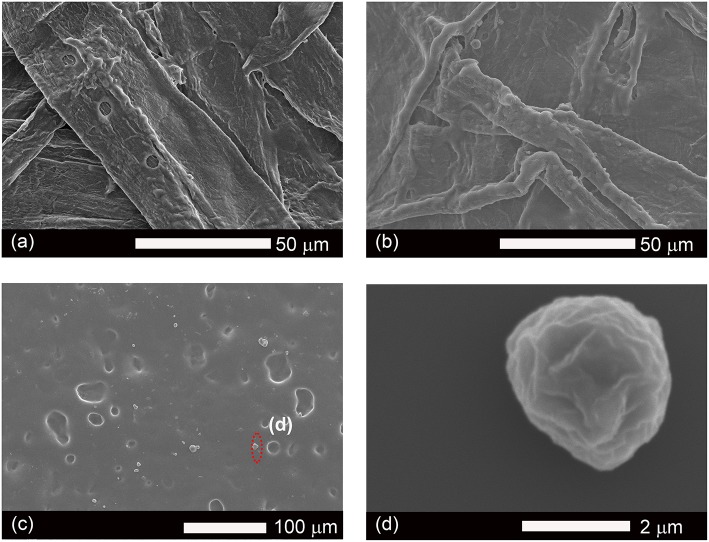
SEM images of surfaces of original paper **(a)**, coated paper **(b)**, coated glass **(c)**, and micro particles of lignin **(d)**.

The morphology of coated surfaces was analyzed by scanning electron microscopy (SEM) and atomic force microscopy (AFM); the roughness of surfaces contributes to the hydrophobic properties of waxy coating materials. Figure 6b showed that the coated surface of pulp boards had a more “uniform” surface than uncoated pulp board (Figure 6a) because the merging of these lignin particles would form a film (Figure 6c) to cover the surface. The AFM showed similar morphology (Figure S6), as particles merged into clusters, seen as linkages amongst droplet islands. This phenomenon resulted from the fact that the samples were stored at room temperature and the coating material has a low glass transition temperature (T_g_ = 8.1°C). At room temperature, the coated lignin particles can soften and relax to the structure of the surface. Similar to latex coatings, once the water has evaporated, those lignin micro particles merged into larger structures, even films at room temperature. Due to the coverage of these hydrophobic lignin particles or films, the coated surface demonstrated improvement on their hydrophobic properties. However, the surface did not retain the microstructured roughness from the dispersed compounds. Hence the roughness resulted from the substrate below the lignin film, which would explain why the smooth glass surface had the lowest contact angle. On the surface of films, there was still a small fraction of lignin particles not fully relaxed (Figure 6d); the diameter for these particles were around 2 μm. This result was in agreement with our previous dynamic light scattering data (Figure 4). Overall, ethyl-oleate lignin coated wood, paper, and glass showed increased hydrophobic properties.

## Conclusion

In this study, a nature inspired lignin-based hydrophobic coating material was developed by esterification of lignin with a plant based fatty acids. The free phenolics were first hydroxyethylated using ethylene carbonate and subsequently, the aliphatic hydroxyls were esterified with oleic acid. Reaction conditions with and without added strong acid as a catalyst provided a derivative with high substitution levels; Utilizing the oleate ethyl-lignin derivative without added acid, lignin micro-particle suspensions were then prepared by controlled precipitation. The oleate HElignin particle showed a particle size centered at 1 μm. By spray or spin coating, this lignin aqueous solution could successfully improve the hydrophobic properties of solid wood and pulp paper with a contact angle above 120°. The low glass transition of the lignin derivative provided the ability of the microparticles to transform on the surface, a dynamic process, conforming to the inherent surface roughness. This renewable coating material helps to advance the field of simple methods to utilize technical lignin in relevant applications for packaging and construction material protection.

## Data Availability

The datasets generated for this study are available on request to the corresponding author.

## Author Contributions

QH, L-YL, and SR designed the experiment. QH and L-YL generated and analyzed the experimental data. The manuscript was written by QH, L-YL, and SR. MK obtained AFM and SEM images and provided analysis. SR has supervised and finalized the final version of the manuscript.

### Conflict of Interest Statement

The authors declare that the research was conducted in the absence of any commercial or financial relationships that could be construed as a potential conflict of interest.

## Supplementary Material

Figure S1 The preparation of aqueous lignin micro particles solution using dialysis to exchange THF and water.

Figure S2 ^31^P NMR spectrum of **(A)** lignin and **(B)** HELignin. Peak (1) internal standard, (2) aliphatic OH groups, (3) aromatic OH groups, 4) COOH groups.

Figure S3 ^13^C NMR spectrum of **(A)** lignin, **(B)** HELignin, **(C)** Oleate esterified HELignin with 1% H_2_SO_4_, and **(D)** Oleate esterified HELignin.

Figure S4 ^1^H NMR spectrum of **(A)** lignin, **(B)** HELignin, and **(C)** oleate HELignin.

Figure S5 GPC traces of oleate HELignin with 1% H_2_SO_4_ as catalysts.

Figure S6 AFM images of aqueous lignin mico particles solution.

Video S1 The surface properties of uncoated pulp.

Video S2 The surface properties of coated pulp.
