# Supplementary material for: Aqueous Dispersions of Esterified Lignin Particles for Hydrophobic Coatings
Source: Front Chem. 2019 Jul 18;7:515. doi: 10.3389/fchem.2019.00515 (PMC6657016; doi:10.3389/fchem.2019.00515)
Supplement: Figure S1 — The preparation of aqueous lignin micro particles solution using dialysis to exchange THF and water. [file Data_Sheet_1.PDF]

Supplementary Material: Aqueous dispersions of esterified lignin particles for hydrophobic coatings

Qi Hua[1], Li-Yang Liu[1], Muzaffer A Karaaslan[1], Scott Renneckar[1] *

[1] Department of Wood Science, The University of British Columbia, Vancouver, BC, Canada

Contact Email: scott.renneckar@ubc.ca

Methods

^{1}H NMR analysis

^{1}H NMR was run to analyze the structural change before and after the modification. Due to the different solubility of resulting lignin, deuterium dimethyl sulfoxide (DMSO-d6) was used to dissolve lignin and HELignin, and toluene-d8 was used for oleate HELignin. 45-50 mg lignin and modified lignin were dissolved into 500 μL corresponding solvent. Above lignin solutions were transferred into 5 mm NMR tubes and tested with a Bruker Avance 300 MHz equipped with a BBO probe at 25 °C. Applied parameters: scan number 256, relaxation delay 10 s, acquisition time 5.30 s, and pulse length 14 μs;

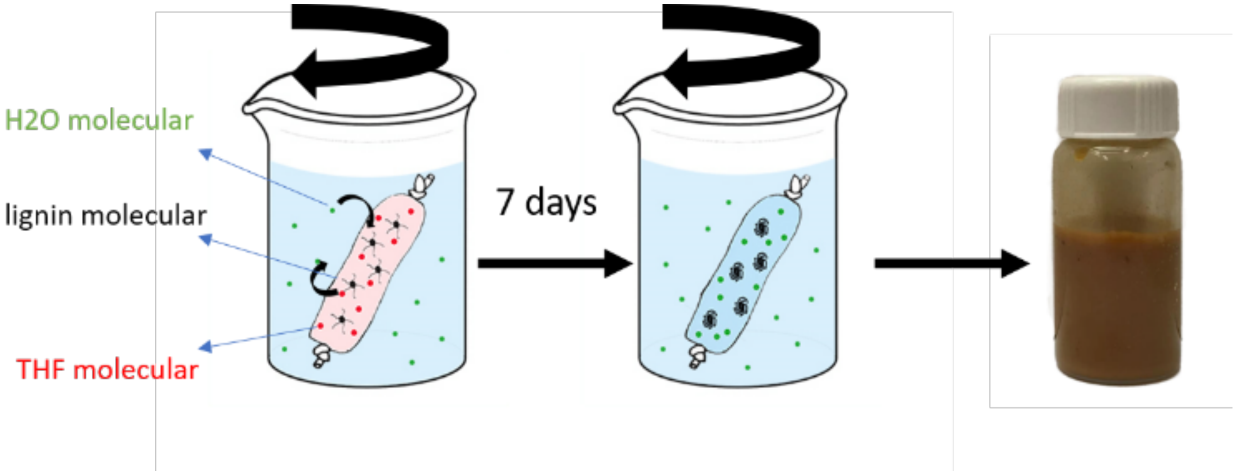

Figure S1 The preparation of aqueous lignin micro-particles solution using dialysis to exchange THF and water

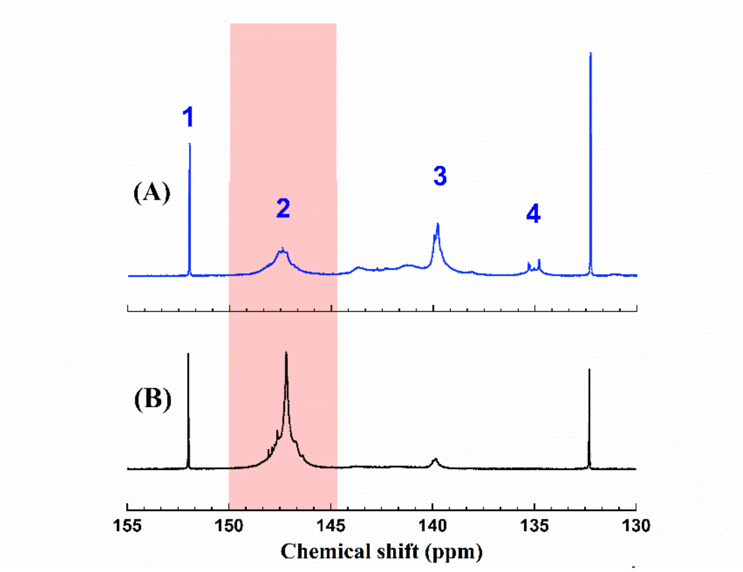

Figure S2 ^{31}P NMR spectrum of (A) lignin and (B) HELignin. Peak 1) internal standard, 2) aliphatic OH groups, 3) aromatic OH groups, 4) COOH groups

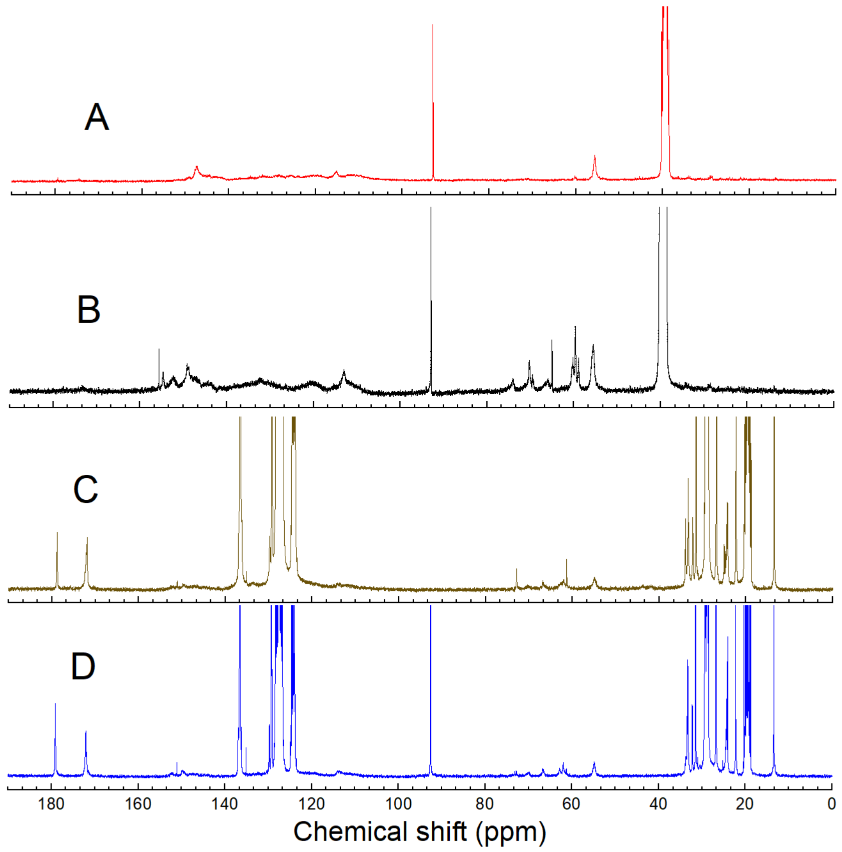

Figure S3 ^{13}C NMR spectrum of A) lignin, B) HELignin, C) Oleate esterified HELignin with 1% H_2SO_4, and D) Oleate esterified HELignin

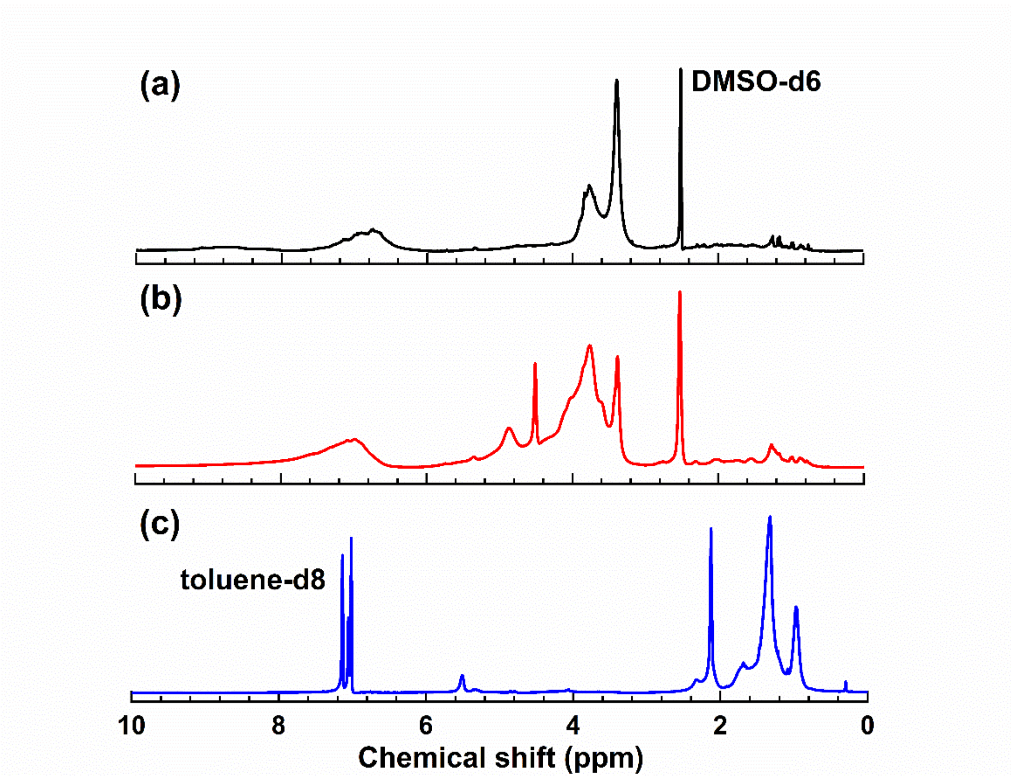

Figure S4 ^{1}H NMR spectrum of (a) lignin, (b) HELignin, and (c) oleate HELignin

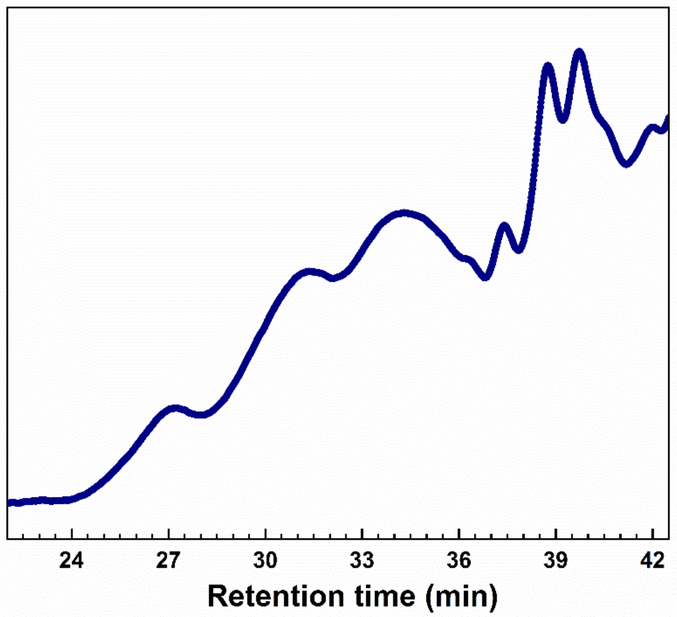

Figure S5 GPC traces of oleate HELignin with 1% H_2SO_4 as catalysts

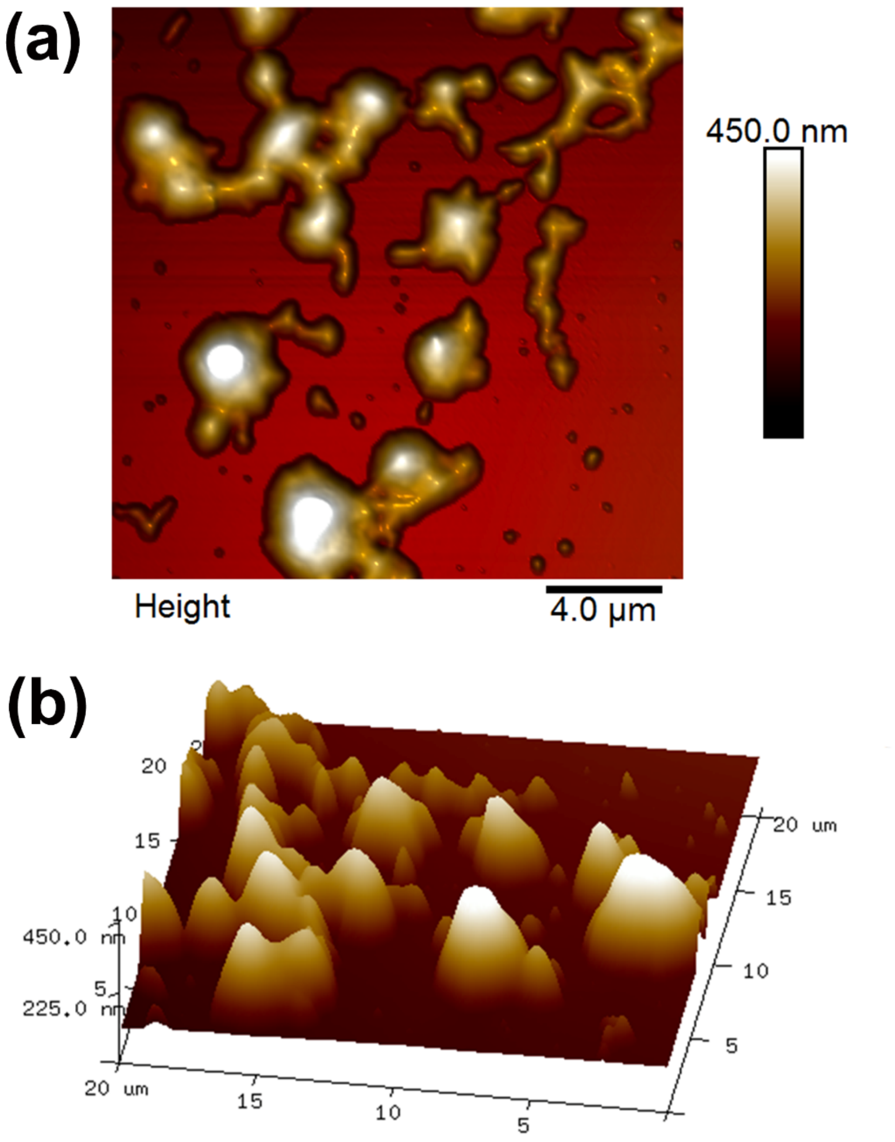

Figure S6 AFM images of aqueous lignin mico particles solution after drying
